# Author Correction: The secreted endoribonuclease ENDU-2 from the soma protects germline immortality in *C. elegans*

**DOI:** 10.1038/s41467-021-23820-7

**Published:** 2021-05-28

**Authors:** Wenjing Qi, Erika D. V. Gromoff, Fan Xu, Qian Zhao, Wei Yang, Dietmar Pfeifer, Wolfgang Maier, Lijiang Long, Ralf Baumeister

**Affiliations:** 1 Bioinformatics and Molecular Genetics (Faculty of Biology), Albert-Ludwigs-University Freiburg, Freiburg, Germany; 2 Spemann Graduate School of Biology and Medicine (SGBM), Albert-Ludwigs-University Freiburg, Freiburg, Germany; 3 Department of Hematology, Oncology and Stem Cell Transplantation, Medical Center-University of Freiburg, Faculty of Medicine, University of Freiburg, Freiburg, Germany; 4 Center for Biochemistry and Molecular Cell Research (ZBMZ, Faculty of Medicine), Albert-Ludwigs-University Freiburg, Freiburg, Germany; 5 Signalling Research Centers BIOSS and CIBSS, Albert-Ludwigs-University Freiburg, Freiburg, Germany

**Keywords:** Cell signalling, Gene expression

Correction to: *Nature Communications* 10.1038/s41467-021-21516-6, published online 24 February 2021.

The original version of this Article contained an error in the author affiliation.

Ralf Baumeister was incorrectly associated with Department of Hematology, Oncology and Stem Cell Transplantation, Medical Center-University of Freiburg, Faculty of Medicine, University of Freiburg, Freiburg, Germany. He is associated with

Bioinformatics and Molecular Genetics (Faculty of Biology), Albert-Ludwigs-University Freiburg, Germany

Spemann Graduate School of Biology and Medicine (SGBM), Albert-Ludwigs-University Freiburg, Germany

Center for Biochemistry and Molecular Cell Research (Faculty of Medicine), Albert-Ludwigs-University Freiburg, Germany

Signalling Research Centers BIOSS and CIBSS, Albert-Ludwigs-University Freiburg, Germany

This has now been corrected in both the PDF and HTML versions of the Article.

