# Supplementary material for: The secreted endoribonuclease ENDU-2 from the soma protects germline immortality in C. elegans
Source: Nat Commun. 2021 Feb 24;12:1262. doi: 10.1038/s41467-021-21516-6 (PMC7904951; doi:10.1038/s41467-021-21516-6)
Supplement: Supplementary file 1 — Supplementary information [file 41467_2021_21516_MOESM1_ESM.pdf]

Supplementary Figure 1

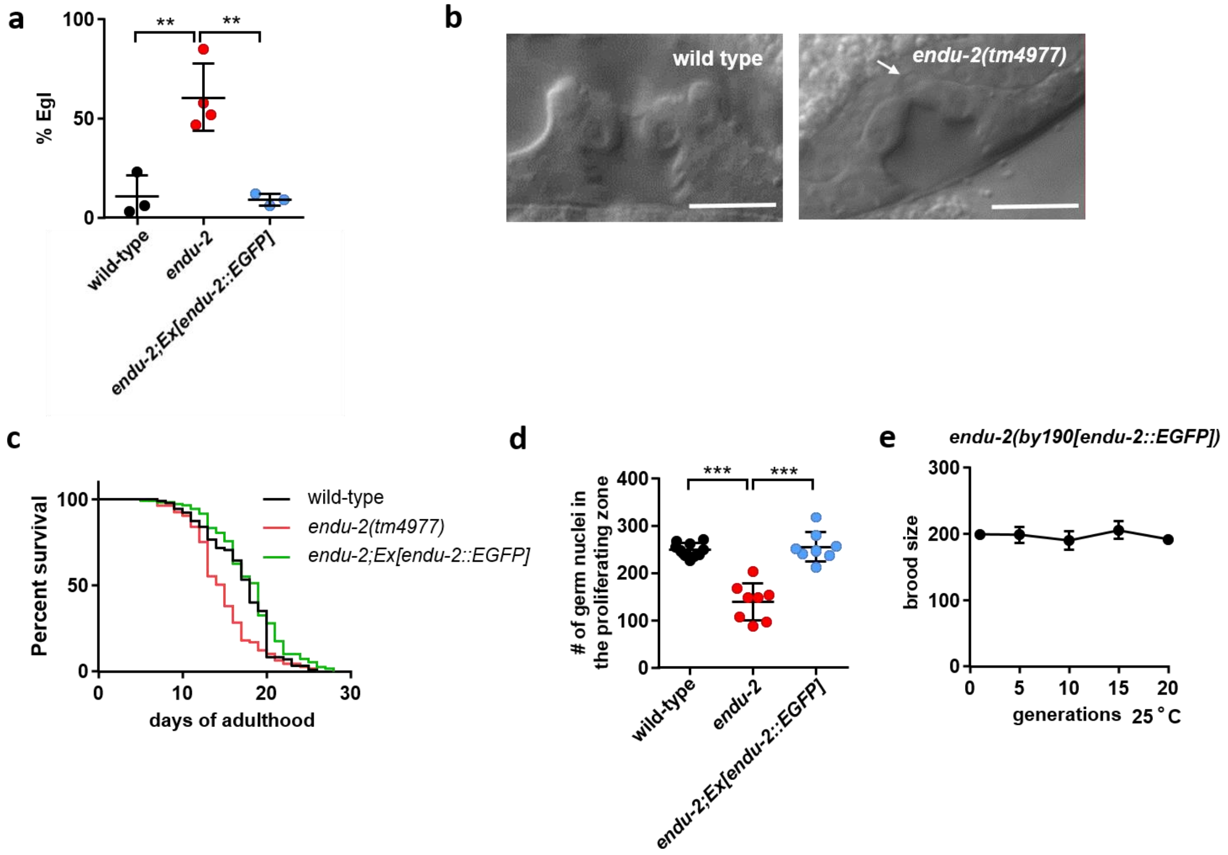

Supplementary Fig. 1: *endu-2* mutants phenotypes and their rescue with extrachromosomal *endu-2::EGFP* transgene.

a) Extrachromosomal *endu-2::EGFP* transgene rescues egg-laying defects (Egl) of *endu-2(lf)* mutants at 20°C. Data are mean ± SD. Number of animals examined over 3 independent experiments n=180 for each strains. Unpaired t test was used to calculate the two-tailed P-values. ** means 0.001 < P-value < 0.01. P-value for *endu-2((tm4977) vs.* wild-type: 0.0069, for *endu-2(tm4977) vs. endu-2;Ex[endu-2::EGFP]*: 0.0038.

b) *endu-2(tm4977)* L4 animals show defective vulva development. The arrow points to the unfused anchor cell. Similar results were obtained in 4 independent experiments. Scale bar: 10 µm.

c) Extrachromosomal *endu-2::EGFP* transgene rescues short lifespan of *endu-2(tm4977)* mutants at 20°C. wild-type: n=83, median survival 18 days, *endu-2(tm4977)*: n=105, median survival 15 days, *endu-2(tm4977);Ex[endu-2::EGFP]*: n=107, median survival 19 days.

d) Extrachromosomal *endu-2::EGFP* transgene rescues reduced germline proliferation of *endu-2(tm4977)* mutants at 20°C. wild-type: n=10, *endu-2(tm4977)*: n=8, *endu-*

2(tm4977);Ex[endu-2::EGFP]: n=8. Data are mean ± SD. Unpaired t test was used to calculate the two-tailed P-values. *** means P-value < 0.0001.

e) The CRISPR EGFP knock-in animals do not show Mrt phenotype at 25°C. Data are mean ± SD. Number of animals examined over 3 independent experiments n=15 for each generation.

All the rescue experiments in this figure were performed with BR7295 *endu-2(tm4977);byEx1375[endu-2P::endu-2::EGFP; myo-2P::mCherry]*.

This Figure is related to the main Figures 1-2.

Supplementary Figure 2

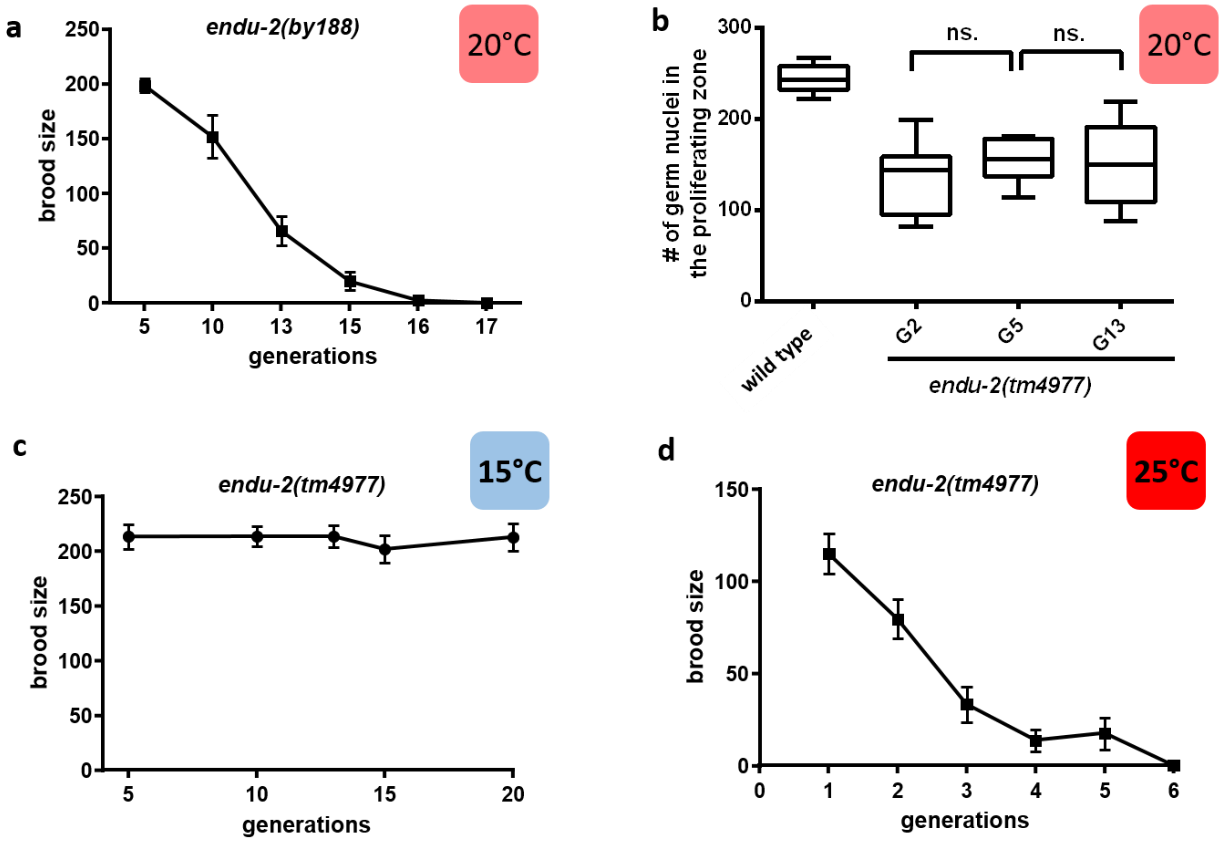

Supplementary Fig. 2: *endu-2(lf)* mutants have a temperature-sensitive Mrt phenotype.

a) *endu-2(by188)* shows gradually reduced brood sizes over generations at 20°C. Data are mean ± SD. Number of examined animals n=11 for G5, n=13 for G10 and G13, n=15 for G15-G17.

b) Germline proliferation of *endu-2(tm4977)* mutants does not change over generations. Shown are boxes extending from the 25th to the 75th percentile, with the median indicated by the horizontal line and Min/Max whiskers. Number of examined animals n=12 for wild type, G2 and G13 of *endu-2(tm4977)*, n=13 for G5 of *endu-2(tm4977)* animals. Unpaired t test was used to calculate the two-tailed P-values. P-value for G5 *vs.* G2: 0.081, G13 *vs* G5: 0.9741.

c) *endu-2(tm4977)* does not exhibit Mrt phenotype at 15°C. Data are mean ± SD. Number of examined animals n=14 for G5, n=15 for G10, n=12 for G13, n=16 for G15 and n=14 for G20.

d) Mrt phenotype of *endu-2(tm4977)* is enhanced at 25°C. Data are mean ± SD. Number of examined animals n=15 for G1 and G2, n=12 for G3, n=15 for G4-G6.

This Figure is related to the main Figure 1.

Supplementary Figure 3

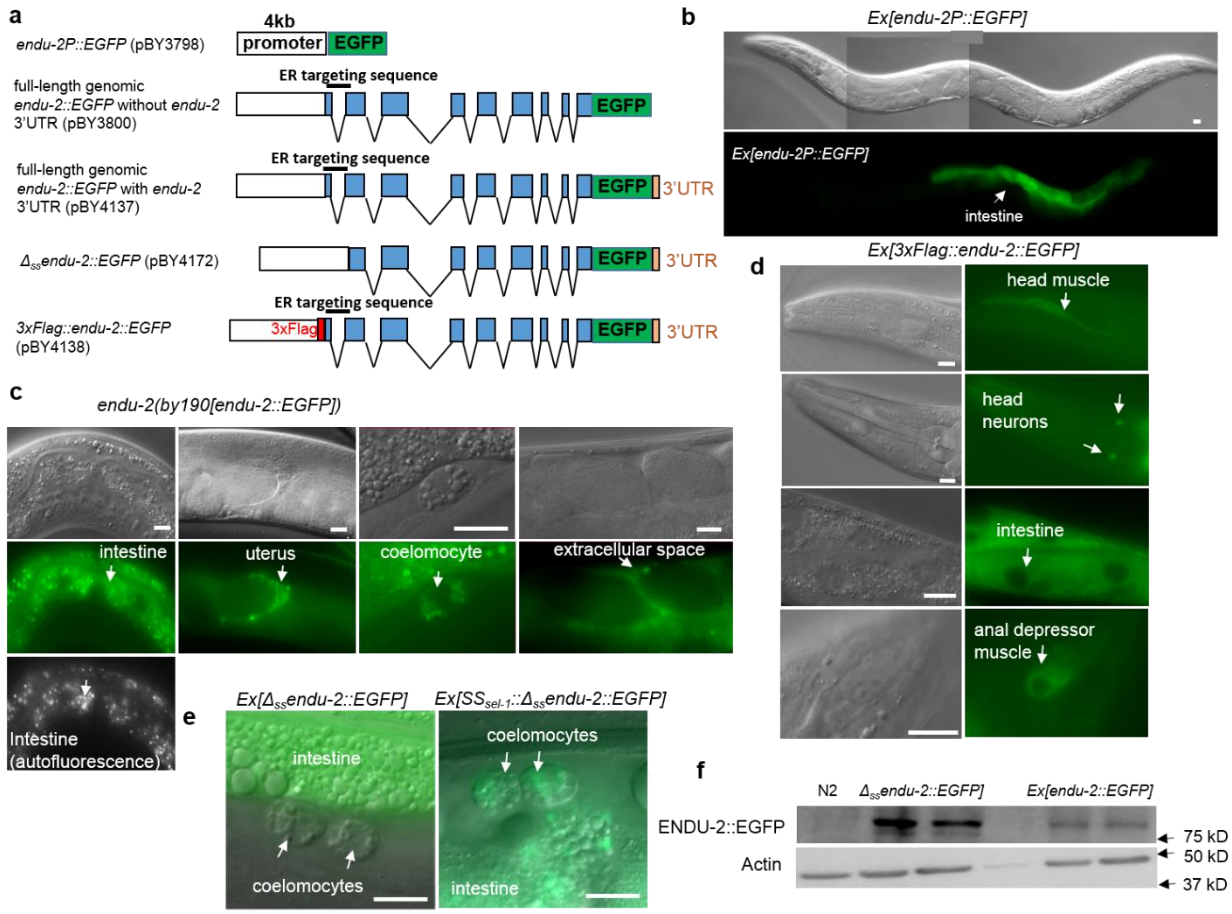

Supplementary Fig. 3: Expression pattern of *endu-2*

a) Summary of the transcriptional and translational fusion reporters of *endu-2* used for study expression and protein localization. All the transgenic strains generated for this study are listed in the Supplementary Data 5.

b) The EGFP transcriptional fusion reporter *byEx1315[endu-2P::EGFP]* shows intestinal expression. Similar expression pattern was observed in 3 independent experiments.

c) The EGFP CRIPSPR-Cas9 knock-in strain *endu-2(by190[endu-2::EGFP])* displays weak ENDU-2::EGFP localization in the intestine, somatic gonad, coelomocyte and extracellular space. Similar expression pattern was observed in more than 5 independent experiments.

d) Localization of 3xFlag::ENDU-2::EGFP (*byEx1805[endu-2P::3xFlag::endu-2::EGFP::* $_{endu\text{-}2}$*3'UTR]* in the intestine, head neurons, muscle cells in the head region and anal depressor muscle. Similar expression pattern was observed in 3 independent experiments.

e) Intestinally expressed Δ_{ss}ENDU-2::EGFP (BR7512 *endu-2(tm4977);byEx1449[endu-2P::Δ_{ss}endu-2::EGFP]*) is not detected in the coelomocyte while fusion of the predicted secretion signal peptide (1-20 amino acids) of SEL-1 to the N-terminal of Δ_{ss}ENDU-2::EGFP (BR8821 *endu-2(tm4977);byEx1875[endu-2P::SS$_{sel-1}$::Δ_{ss}endu-2::EGFP]*) results in its localization in the coelomocytes. Similar expression pattern was observed in 3 independent experiments.

f) Western Blot to detect localization of transgenically expressed ENDU-2(wt)::EGFP and Δ_{ss}ENDU-2::EGFP. Shown are two replicates with 10 day one adult animals of BR8657 *endu-2(tm4977);byEx1814[endu-2P::endu-2::EGFP::$_{endu-2}$3'UTR]* and BR8747 *endu-2(tm4977);byEx1847[endu-2P::Δ_{ss}endu-2::EGFP::$_{endu-2}$3'UTR]* at 25°C. Wild type animals were used as negative control. Similar results were observed in three biological replicates. The uncropped blots are included in the Supplementary Fig. 11a.

Scale bar 10 µm. This Figure is related to the main Figure 2.

Supplementary Figure. 4

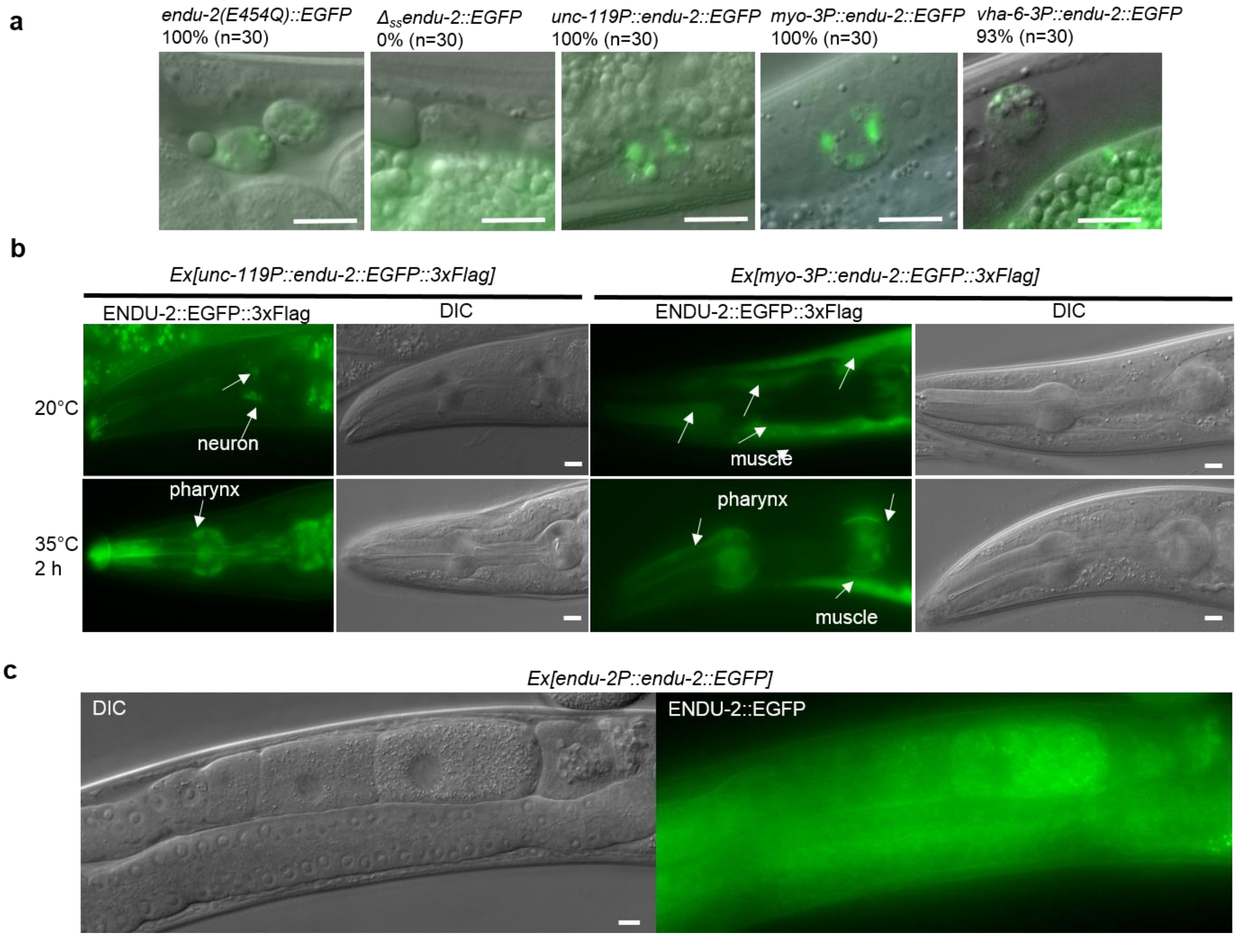

Supplementary Fig. 4: ENDU-2::EGFP is a secreted protein

a) Accumulation of secreted ENDU-2::EGFP in the coelomocytes. N=3 biological replicates.

b) Heat stress leads to increased pharyngeal accumulation of ENDU-2::EGFP expressed from neurons and muscle cells. N=3 biological replicates.

c) Extra-chromosomal *endu-2::EGFP* transgene results in a weak ENDU-2::EGFP signal in the oocytes (9%, n=100). N=4 biological replicates.

Scale bar 10 µm. This Figure is related to the main Figure 2.

Supplementary Figure 5

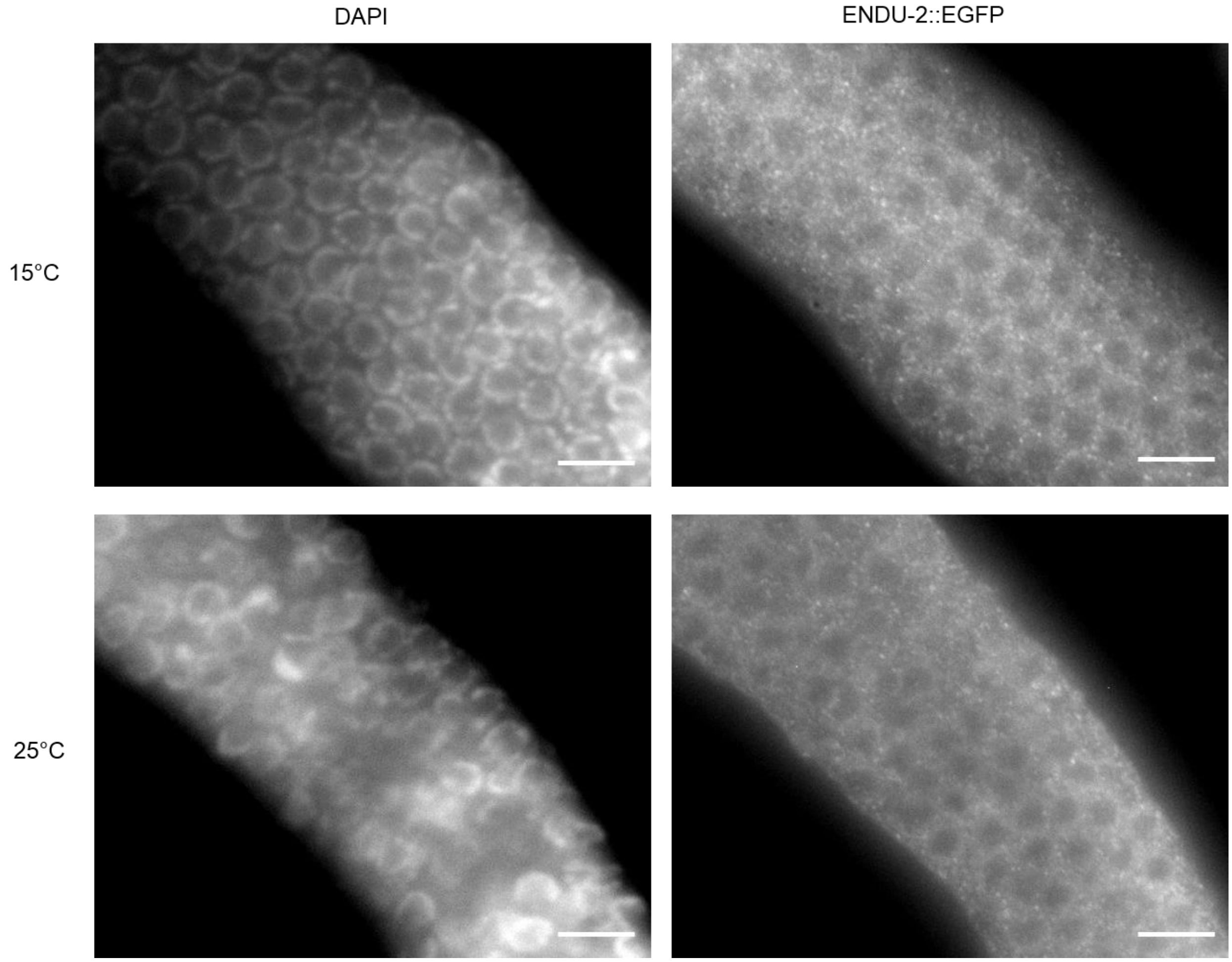

Supplementary Fig. 5: ENDU-2::EGFP is localized in the germline at both 15°C and 25°C.

ENDU-2::EGFP in *endu-2(by190[endu-2::EGFP])* was detected with GFP antibody. Scale bar 10 µm. N=3 replicates

This Figure is related to the main Figure 2.

Supplementary Figure 6

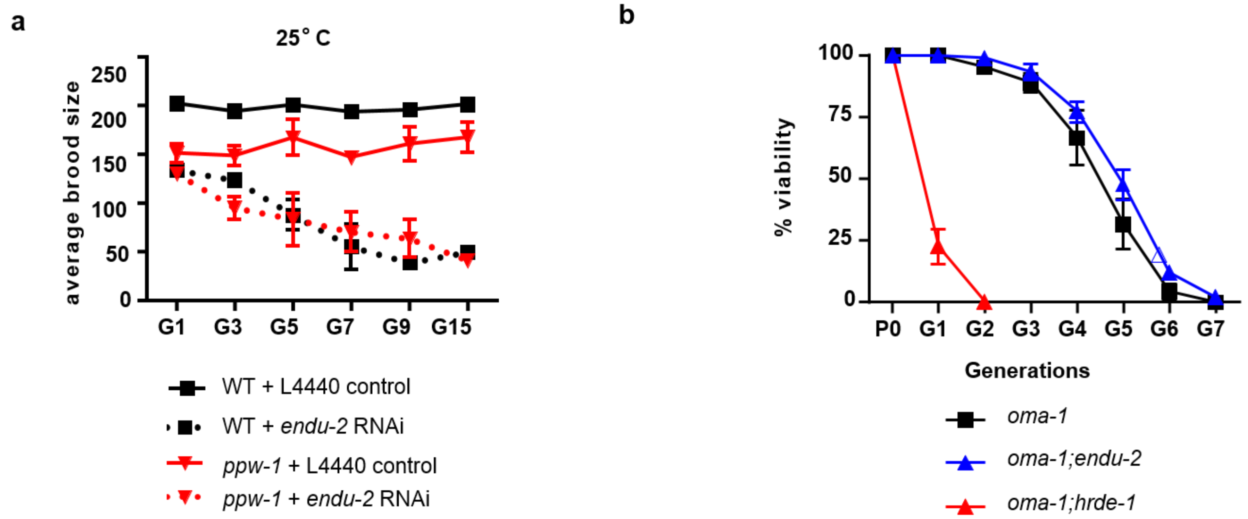

Supplementary Fig. 6: Somatically expressed ENDU-2 affects reproduction without affecting multigenerational inheritance of *oma-1* RNAi in the germline.

a) *endu-2* RNAi causes gradually reduced brood size in both wild type and *ppw-1(pk1425)* animals. Data are mean ± SD. Number of animals examined over 3 independent experiments n=15 for each generation.

b) *endu-2(tm4977)* mutant animals are not defective in multigenerational inheritance of *oma-1* RNAi in the germline. Data are mean ± SEM. Numbers of examined embryos over three independent experiments: *oma-1(zu405):* G1 n=316, G2 n=315, G3 n=346, G4 n=376, G5 n=412, G6 n=612, *oma-1(zu405);endu-2(tm4977)*: G1 n=336, G2 n=307, G3 n=360, G4 n=323, G5 n=335, G6 n=334, G7 n=492, *oma-1(zu405);hrde-1(tm1200)*: G1 n=335, G2 n=206.

This Figure is related to the main Figure 3.

Supplementary Figure 7

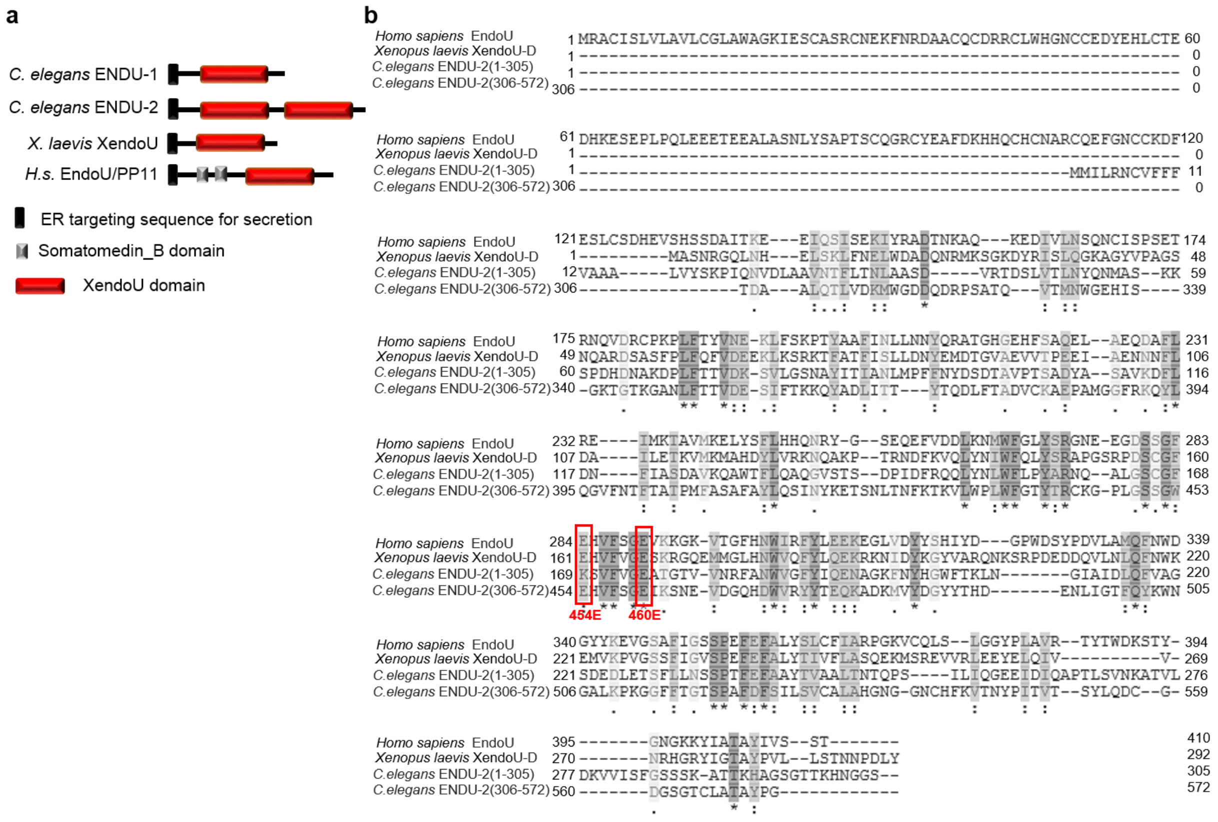

Supplementary Fig. 7: ENDU-2 is an evolutionarily conserved endoribonuclease

a) Protein domain prediction of EndoU in *C. elegans*, *X. laevis* and *H. sapiens*.

b) Sequence alignment of human EndoU, *Xenopus* XendoU and *C. elegans* ENDU-2 suggests sequence conservation of the XendoU domains from worm to human. As *C. elegans* ENDU-2 has two XendoU domains, the alignment was performed with split protein fragments containing one of the XendoU domains (1-305 and 306-572 amino acids) each.

This Figure is related to the main Figure 4.

Supplementary Figure 8.

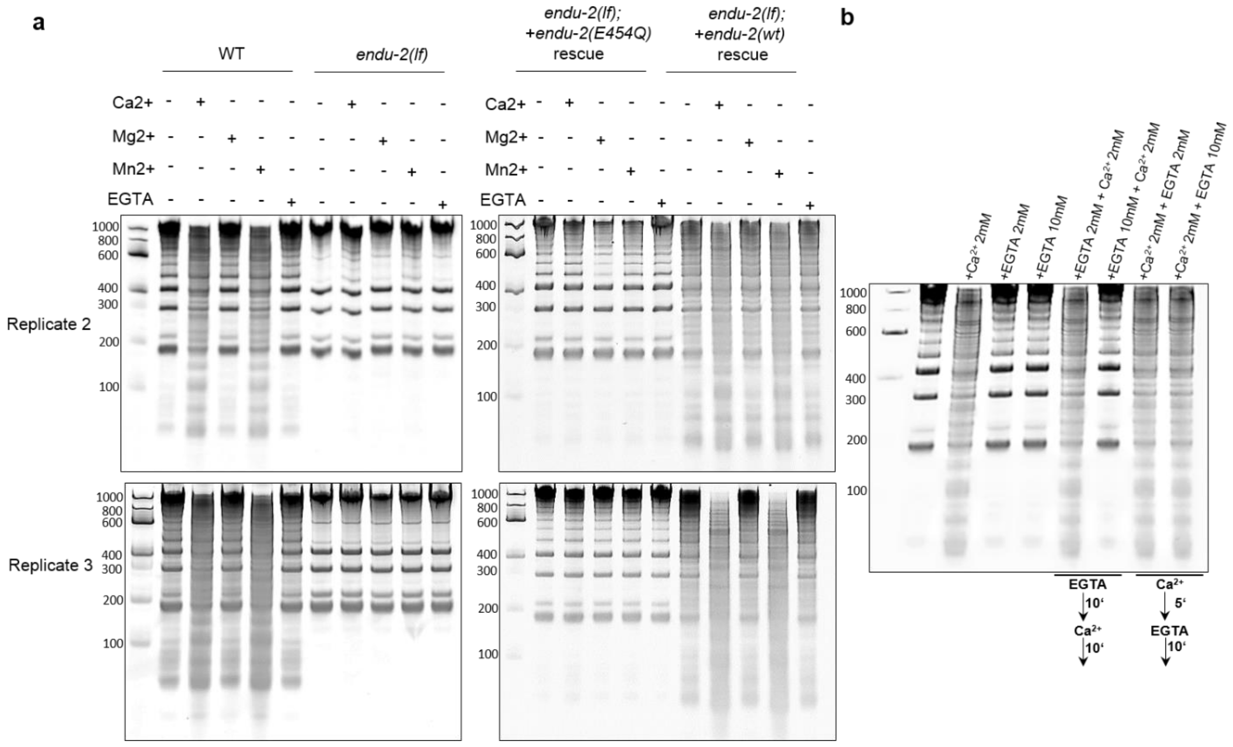

Supplementary Fig. 8: Ca^{2+} and Mn^{2+} promote ENDU-2 mediated RNA decay.

a) Two additional biological replicates showing ENDU-2 mediated bulk RNA decay in the presence of Ca^{2+} and Mn^{2+}. N=3 independent experiments.

b) Incubation with Ca^{2+} results in decay of total RNA isolated from wild type animals. This effect is blocked by pre-incubation with EGTA. N=3 independent experiments.

This Figure is related to the main Figure 4.

Supplementary Figure 9.

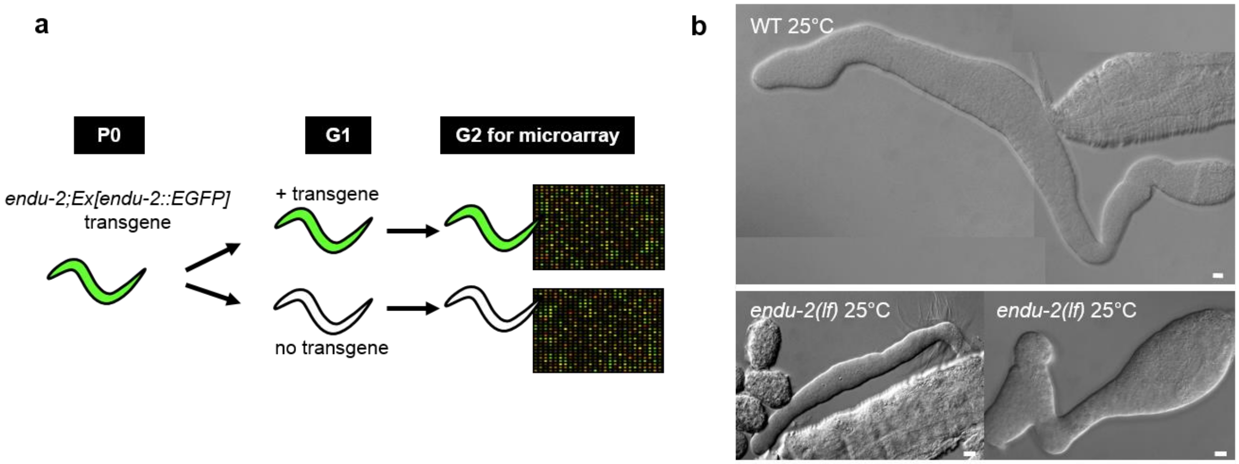

Supplementary Fig. 9: Transcriptomic analysis with microarray

a) Schematic diagram of strain preparations for microarray analyses. The G2 granddaughter generation from one single *endu-2(lf);Ex[endu-2::EGFP]* was used for transcriptome analysis.

b) Gonad of *endu-2(lf)* mutants are smaller than wild type animals at 25°C. Scale bar 10 µm. N=3 independent replicates.

This Figure is related to the main Figure 5.

Supplementary Figure 10.

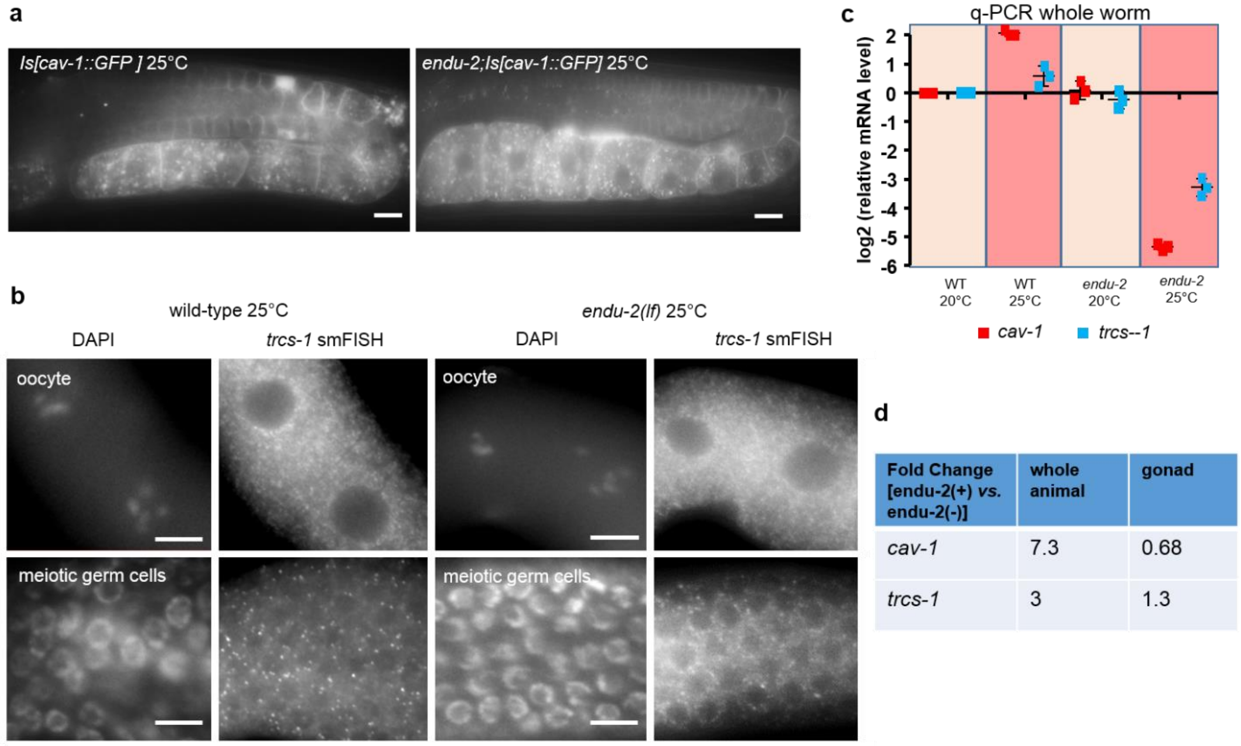

d

Fold Change [endu-2(+) *vs.* endu-2(-)]	whole animal	gonad
cav-1	7.3	0.68
trcs-1	3	1.3

Supplementary Fig. 10: Comparison of different assays to determine expression levels of ENDU-2 binding targets *cav-1* and *trcs-1* in the germline.

a) ENDU-2 does not significantly affect the expression level of *cav-1::GFP* in the germline. N=3 independent replicates.

b) smFISH staining reveals no significant fold change of *trcs-1* mRNA in the germline upon *endu-2* knock-down. N=3 independent replicates.

c) q-PCR results suggest strongly decreased mRNA level of both *trcs-1* and *cav-1* in the absence of *endu-2* at elevated temperature. Data are mean ± SD.

d) Transcriptomic studies reveal distinct *cav-1* and *trcs-1* mRNA levels from RNA extractions of either whole animal or gonad-only origin.

Scale bar 10 µm. This Figure is related to the main Figure 5.

Supplementary Figure 11.

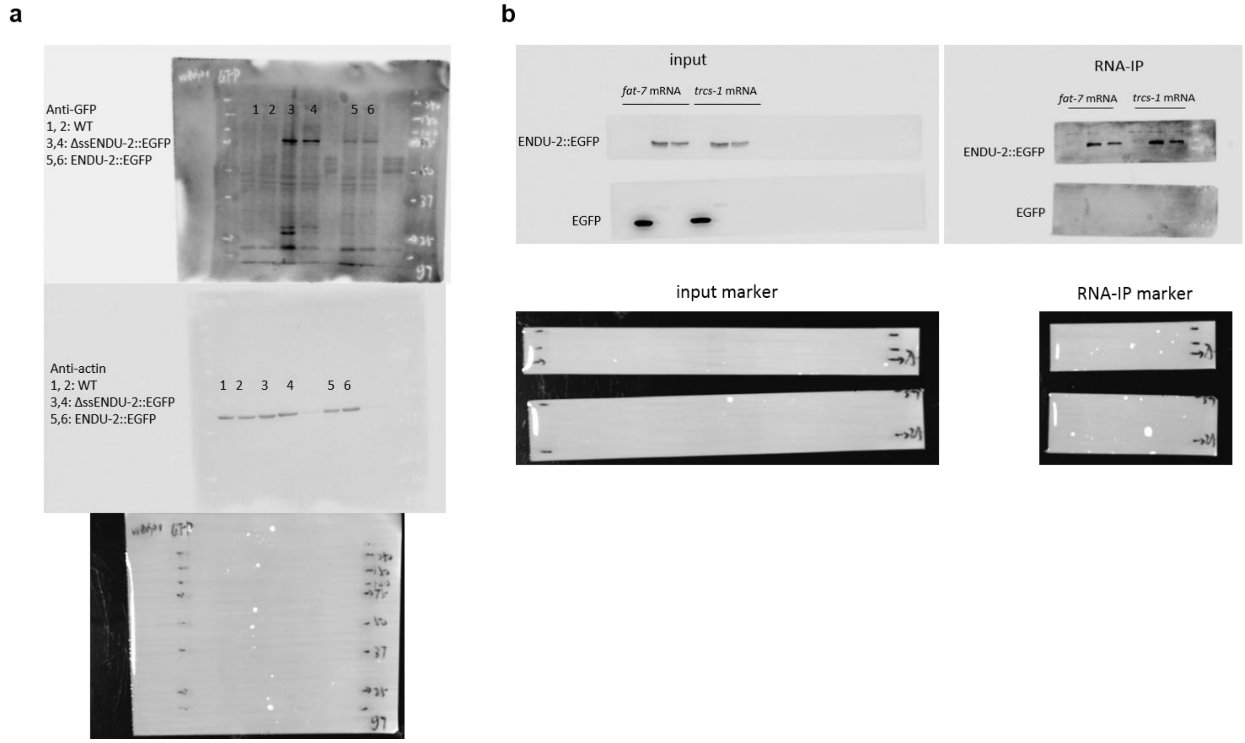

Supplementary Fig. 11: Uncropped blots for Western blots in this study.

a) Uncropped blots for the Supplementary Fig. 3f.

b) Uncropped blots for the Fig. 4c.
