# Supplementary material for: The secreted endoribonuclease ENDU-2 from the soma protects germline immortality in C. elegans
Source: Nat Commun. 2021 Feb 24;12:1262. doi: 10.1038/s41467-021-21516-6 (PMC7904951; doi:10.1038/s41467-021-21516-6)
Supplement: Supplementary file 7 — Reporting Summary [file 41467_2021_21516_MOESM7_ESM.pdf]

natureresearch

Corresponding author(s): Wenjing Qi, PhD

Last updated by author(s): Dec 31, 2020

Reporting Summary

Nature Research wishes to improve the reproducibility of the work that we publish. This form provides structure for consistency and transparency in reporting. For further information on Nature Research policies, see Authors & Referees and the Editorial Policy Checklist .

Statistics

For all statistical analyses, confirm that the following items are present in the figure legend, table legend, main text, or Methods section.

n/a	Confirmed	
☐	☒	The exact sample size (*n*) for each experimental group/condition, given as a discrete number and unit of measurement
☐	☒	A statement on whether measurements were taken from distinct samples or whether the same sample was measured repeatedly
☐	☒	The statistical test(s) used AND whether they are one- or two-sided<br>*Only common tests should be described solely by name; describe more complex techniques in the Methods section.*
☐	☒	A description of all covariates tested
☐	☒	A description of any assumptions or corrections, such as tests of normality and adjustment for multiple comparisons
☐	☒	A full description of the statistical parameters including central tendency (e.g. means) or other basic estimates (e.g. regression coefficient) AND variation (e.g. standard deviation) or associated estimates of uncertainty (e.g. confidence intervals)
☐	☒	For null hypothesis testing, the test statistic (e.g. *F*, *t*, *r*) with confidence intervals, effect sizes, degrees of freedom and *P* value noted<br>*Give P values as exact values whenever suitable.*
☒	☐	For Bayesian analysis, information on the choice of priors and Markov chain Monte Carlo settings
☒	☐	For hierarchical and complex designs, identification of the appropriate level for tests and full reporting of outcomes
☒	☐	Estimates of effect sizes (e.g. Cohen's *d*, Pearson's *r*), indicating how they were calculated

Our web collection on statistics for biologists contains articles on many of the points above.

Software and code

Policy information about availability of computer code

Data collection	No
Data analysis	Tissue enrichment and GO term enrichment analysis was carried out with the online enrichment analysis tool (https://wormbase.org/tools/enrichment/tea/tea.cgi)

For manuscripts utilizing custom algorithms or software that are central to the research but not yet described in published literature, software must be made available to editors/reviewers. We strongly encourage code deposition in a community repository (e.g. GitHub). See the Nature Research guidelines for submitting code & software for further information.

Data

Policy information about availability of data

All manuscripts must include a data availability statement. This statement should provide the following information, where applicable:

- Accession codes, unique identifiers, or web links for publicly available datasets
- A list of figures that have associated raw data
- A description of any restrictions on data availability

Provide your data availability statement here.

Field-specific reporting

Please select the one below that is the best fit for your research. If you are not sure, read the appropriate sections before making your selection.

☒ Life sciences ☐ Behavioural & social sciences ☐ Ecological, evolutionary & environmental sciences

Life sciences study design

All studies must disclose on these points even when the disclosure is negative.

Sample size	We chose sample sizes that have been wildly accepted in the field for phenotype analysis . For RIP-seq experiment we performed three GFP control IP to address which RNAs are false positive RNA targets of ENDU-2. Four biological replicates for ENDU-2(wt) RIP-seq and nine biological replicates for ENDU-2(E454Q) RIP-seq. Three biological replicates for microarray study and two biological replicates for gonadal RNA-seq.
Data exclusions	Animals that had died due to internal hatching were censored from the lifespan assay.
Replication	All attempts at replication were successful.
Randomization	Samples were allocated randomly into experimental groups.
Blinding	The lifespan assay and scoring germline phenotype were not performed blindly as the obvious mutant phenotypes make blinding surplus.

Reporting for specific materials, systems and methods

We require information from authors about some types of materials, experimental systems and methods used in many studies. Here, indicate whether each material, system or method listed is relevant to your study. If you are not sure if a list item applies to your research, read the appropriate section before selecting a response.

Materials & experimental systems

n/a	Involved in the study	
☐	☒	Antibodies
☐	☒	Eukaryotic cell lines
☒	☐	Palaeontology
☐	☒	Animals and other organisms
☒	☐	Human research participants
☒	☐	Clinical data

Methods

n/a	Involved in the study	
☒	☐	ChIP-seq
☒	☐	Flow cytometry
☒	☐	MRI-based neuroimaging

Antibodies

Antibodies used	anti-GFP antibody ChIP grade ab290 (Abcam)
Validation	Rabbit polyclonal to GFP - ChIP Grade<br>https://www.abcam.com/gfp-antibody-chip-grade-ab290.html

Eukaryotic cell lines

Policy information about cell lines

Cell line source(s)	HEK293 cells
Authentication	The HEK293 cell line was not authenticated as the cells were used for protein expression.
Mycoplasma contamination	All the cell lines were negative for mycoplasma contamination
Commonly misidentified lines<br>(See ICLAC register)	No

Animals and other organisms

Policy information about studies involving animals; ARRIVE guidelines recommended for reporting animal research

Laboratory animals	Caenorhabditis elegans
Wild animals	No
Field-collected samples	No

Ethics oversight	No ethical approval was required as all the experiments were performed in C. elegans or in HEK293 cells.

Note that full information on the approval of the study protocol must also be provided in the manuscript.
