# Supplementary material for: The secreted endoribonuclease ENDU-2 from the soma protects germline immortality in C. elegans
Source: Nat Commun. 2021 Feb 24;12:1262. doi: 10.1038/s41467-021-21516-6 (PMC7904951; doi:10.1038/s41467-021-21516-6)
Supplement: Supplementary file 9 — Description of Additional Supplementary Files [file 41467_2021_21516_MOESM9_ESM.pdf]

Title: Supplementary Data 1.
Description: Summary of RIP-seq results Description: Summary of the transcripts that co-immunoprecipitate with ENDU-2(E454Q)::EGFP.

Title: Supplementary Data 2.
Description: Summary of microarray result Description: Differentially expressed genes in *endu-2(tm4977) vs. endu-2(tm4977); byEx1375[endu2P::endu-2::EGFP]* at 25°C.

Title: Supplementary Data 3.
Description: Summary of the gonadal RNA-Seq data. Description: List of genes expressed in the wild type gonad and differentially expressed genes in gonad of *endu-2(tm4977) vs.* wild type at 25°C.

Title: Supplementary Data 4.
Description: Summary of smFISH probes. Description: Sequences of the smFISH probes to detect *endu-2, fat-7* and *trcs-1* mRNA.

Title: Supplementary Data 5.
Description: Transgenic strains generated in this study. Description: Summary of all the transgenic animals generated for this study.
